# Animal Models of Diabetes and Metabolic Disease

**DOI:** 10.1155/2013/281928

**Published:** 2013-06-25

**Authors:** Tomohiko Sasase, Marcus G. Pezzolesi, Norihide Yokoi, Takahisa Yamada, Kozo Matsumoto

**Affiliations:** ^1^Biological/Pharmacological Research Laboratories, Central Pharmaceutical Research Institute, Japan Tobacco Inc., Osaka 569-1125, Japan; ^2^Section on Genetics and Epidemiology, Joslin Diabetes Center, Harvard Medical School, Boston, MA 02215, USA; ^3^Division of Molecular and Metabolic Medicine, Department of Physiology and Cell Biology, Kobe University Graduate School of Medicine, Kobe 650-0017, Japan; ^4^Laboratory of Animal Genetics, Division of Life and Food Sciences, Graduate School of Science and Technology, Niigata University, Niigata 950-2181, Japan; ^5^Department of Laboratory Animal Science, Faculty of Life Sciences, Kyoto Sangyo University, Kyoto 603-8555, Japan

Metabolic diseases, including diabetes (and its complications), obesity, dyslipidemia, and hypertension, are common diseases and frequently occur in combination. To study these diseases, many hereditary animal models have been reported, such as *ob/ob* mouse, *db/db* mouse, KK-Ay mouse, Goto-Kakizaki (GK) rat, Zucker diabetic fatty (ZDF) rat, Otsuka-Long-Evans-Tokushima-fatty (OLETF) rat, Spontaneously Diabetic Torii (SDT) rat, spontaneously hypertensive rat (SHR), Watanabe Heritable hyperlipidemic (WHHL) rabbit, and Postprandial hypertriglyceridemia (PHT) rabbit. Chemical (e.g., streptozotocin (STZ), alloxan) and diet-induced (e.g., high sucrose, high fat, high cholesterol) experimental animal models have been in general use for a long time, and genetically modified animals have also been used widely in recent decades. Although molecular biological techniques have become more important for clarifying the mechanisms of the diseases, the importance of animal models has not changed. Animal models are needed to reveal the underlying pathophysiology of metabolic diseases; this approach provides information that is the key to the development of new therapies and drugs to treat and manage these diseases. In this special issue, we aim to provide information on recent beneficial experimental animal models in this field and present up-to-date information on the pathophysiology, therapeutic drugs, and diagnosis of metabolic diseases using valuable animal models. We believe that the 14 articles in this special issue satisfy these aims.

Type 2 diabetes (T2D) is a complex, multifactorial disease. Both genetic and environmental factors are known to contribute to its development; however, the precise pathogenesis of T2D remains largely unclear. In the present special issue, various spontaneous T2D rodent models are summarized in *“Spontaneous type 2 diabetic rodent models”* by Y.W. Wang et al. 

Several new animal models are introduced in this special issue. Three papers introduce new diabetic animal models, SDT rat and its derivative SDT fatty rat. The SDT rat, a nonobese T2D model, shows severe hyperglycemia accompanied with hypoinsulinemia. In the review article *“The Spontaneously Diabetic Torii rat: an animal model of nonobese type 2 diabetes with severe diabetic complications”* by T. Sasase et al., characteristics of diabetic complications in SDT rats, such as diabetic retinopathy, diabetic peripheral neuropathy, diabetic neuropathy, and osteoporosis, are described. Among these diabetic complications, ocular complications are notably a unique characteristic of this animal model. Using this model, A. Ota et al. evaluated ranirestat, a novel aldose reductase inhibitor that is currently in phase III clinical trial, on diabetic complications, and report their findings in *“Effects of long-term treatment with ranirestat, a potent aldose reductase inhibitor, on diabetic cataract and neuropathy in Spontaneously Diabetic Torii rats.”* The SDT fatty rat is an obese T2D model established by introducing the *fa * allele of Zucker fatty rat into the SDT rat genome. In the review article *“Metabolic disorders and diabetic complications in Spontaneously Diabetic Torii Lepr* ^*fa*^ *rat: a new obese type 2 diabetic model,”* Y. Kemmochi et al. review the pathophysiological features of this animal model.

In *“A novel rat model of type 2 diabetes: the Zucker fatty diabetes mellitus ZFDM rat,”* N. Yokoi et al. report the establishment and characteristics of ZFDM rat. This new T2D model is derived from the ZF rat, presents with hyperglycemia, and is phenotypically distinct from normoglycemic ZF rats. Loss of islet architecture and fibrosis are also observed in the ZFDM rat. This model is useful for investigating young- to middle-aged adult-onset T2D.

T. Okamura et al. report the establishment and characteristics of Long-Evans Agouti (LEA) rat derived from Long-Evans (LE) strain, in *“Phenotypic characterization of LEA rat: a new rat model of nonobese type 2 diabetes.”* The LEA rat displays moderate hyperglycemia and glucose intolerance along with impaired insulin secretion that is caused by progressive fibrosis in pancreatic islets. This model is useful in investigations on the progression of T2D with age.

Three articles describe genetic factors of spontaneous T2D models, in detail. In *“Genetic dissection of complex genetic factor involved in NIDDM of OLETF rat,”* T. Yamada et al. review 14 QTLs (*Nidd1–14/of*) identified in OLETF rat that are responsible for noninsulin dependent diabetes mellitus (NIDDM). Their work showed that NIDDM in the OLETF rat is a highly heterogeneous and complex genetic disorder. In *“Single diabetic QTL derived from OLETF rat is a sufficient agent for severe diabetic phenotype in combination with leptin-signaling deficiency,”* H. Kose et al. map hyperglycemia QTLs in the OLETF rat and show that *Nidd2/of *is highly responsive to obesity. In addition, *Nidd2/of* and another hyperglycemia QTL, *Nidd1/of,* appear to act in combination to lead to the development of diabetes. Their new congenic strain, carrying a single QTL, may be helpful in identifying the causative gene and should aid future investigations on understanding the mechanism by which obesity interacts with QTLs to regulate diabetic traits.

In the article *“Dose effect and mode of inheritance of diabetogenic gene on mouse chromosome 11,”* N. Babaya et al. revealed that the introgression of chromosome 11 of T2D Nagoya-Shibata-Yasuda (NSY) strain onto nondiabetic C3H caused marked changes in glucose tolerance and STZ susceptibility, even in heterozygous state. However, hyperglycemia and STZ sensitivity in heterozygous C3H-11^NSY^ mouse was not as severe as in the homozygous mouse. The consomic strains constructed in their studies will facilitate fine mapping and identification of responsible genes for T2D-related phenotypes.

Islet transplantation is an effective treatment for severe diabetes. N. Sakata et al. review three types of animal models (STZ-induced diabetes, pancreatomized diabetes, and spontaneous diabetes) used for preclinical islet transplantation research in *“Animal models of diabetes mellitus for islet transplantation.”* As highlighted in this review, selecting the appropriate models based on the study's objective is paramount to properly interpret a study's findings and critical to advance this area of research.

Cardiovascular disorders (CVDs) are the leading cause of death in humans. In *“Cardiovascular changes in animal models of metabolic syndrome”* by A. M. Lehnen et al., cardiovascular findings in animal models with obesity, dyslipidemia, diabetes, or hypertension are summarized. The use of different experimental models is important in order to better understand the mechanisms involved in CVDs caused by metabolic disease.

Because the mortality due to CVD is greater in type 1 diabetic (T1D) patients than in the general population, C. H. Hung et al. aimed to investigate the severity of cardiopulmonary dysfunction caused by lipopolysaccharide (LPS) in T1D rats in *“Cardiopulmonary profile in streptozotocin-induced type 1 diabetic rats during systemic endotoxemia.”* In this study, despite higher TNF-*α* level in serum and bronchoalveolar lavage fluid between nondiabetic and STZ T1D rats, there was only a modest difference of cardiopulmonary dysfunction between the two groups.

T1D is an autoimmune disease that is characterized by the selective destruction of pancreatic *β* cells. Both environmental and genetic factors are known to contribute to the progression of this autoimmunity. In the review article *“Rodent models for investigating the dysregulation of immune responses in type 1 diabetes”* by F. C. Chou et al., non-obese diabetic (NOD) mouse and other spontaneous diabetes models are introduced. In addition, this review discusses genetically manipulated NOD mice that overexpress protective genes in islet, T-cell receptors, islet-specific neoantigens, or humanized MHC.

Lastly, in their review article entitled *“Foxp3* ^+^ *regulatory T cells in mouse models of type 1 diabetes,” *C. Petzold et al. focus on a murine model of T1D that expresses the Foxp3^+^ transcription factor in CD4^+^ CD25^+^ regulatory T (Treg) cells. In this review, spontaneous and genetically engineered NOD mice for studying the role of Foxp3^+^ Treg cells in *β* cell autoimmunity are overviewed.

*Tomohiko Sasase* *Tomohiko Sasase* *Marcus G. Pezzolesi* *Marcus G. Pezzolesi* *Norihide Yokoi* *Norihide Yokoi* *Takahisa Yamada* *Takahisa Yamada* *Kozo Matsumoto* *Kozo Matsumoto*

